# Incidence of type 2 diabetes by socioeconomic deprivation in Germany between 2014 and 2019: an ecological study

**DOI:** 10.1136/bmjopen-2024-094824

**Published:** 2025-07-16

**Authors:** Katharina Piedboeuf-Potyka, Ramona Hering, Mandy Schulz, Malwina Mackowiak, Ralph Brinks, Oliver Kuß, Annika Hoyer, Thaddäus Tönnies

**Affiliations:** 1 Institute for Biometry and Epidemiology, German Diabetes Center Leibniz Center for Diabetes Research at the Heinrich Heine University Düsseldorf, Düsseldorf, Nordrhein-Westfalen, Germany; 2 Department of Healthcare Analyses, Central Research Institute of Ambulatory Health Care, Berlin, Germany; 3 Department of Mathematics, Informatics, Technology, University of Applied Sciences Koblenz, Koblenz, Rheinland-Pfalz, Germany; 4 Chair for Medical Biometry and Epidemiology (MBE), Witten/Herdecke University, Faculty of Health/School of Medicine, Witten/Herdecke, Germany; 5 Centre for Health and Society, Medical Faculty and University Hospital Düsseldorf, Heinrich-Heine-Universitat Dusseldorf, Düsseldorf, Germany; 6 German Center for Diabetes Research, München-Neuherberg, Germany; 7 Medical School OWL, Biostatistics and Medical Biometry, Bielefeld University, Bielefeld, Germany

**Keywords:** Diabetes Mellitus, Type 2, PUBLIC HEALTH, EPIDEMIOLOGY, Health Equity

## Abstract

**Abstract:**

**Objective:**

To estimate type 2 diabetes incidence trends by sex and socioeconomic position (SEP) and evaluate trends in SEP-related inequalities in incidence.

**Design:**

Ecological study using ambulatory claims data and regression-based modelling.

**Setting:**

All 401 counties in Germany, covering the entire country.

**Participants:**

All individuals with statutory health insurance (~85% of the population). Incident cases of type 2 diabetes were identified annually from 2014 to 2019 using the International Statistical Classification of Diseases and Related Health Problems, 10th revision codes.

**Primary and secondary outcome measures:**

Incident type 2 diabetes at the county level, adjusted for age and modelled using a mixed negative binomial regression. SEP was measured using the German Index of Socioeconomic Deprivation, and a random intercept accounted for county-level heterogeneity.

**Results:**

The incidence of type 2 diabetes decreased between 2014 and 2017 and plateaued thereafter. Trends were similar between sexes and deprivation levels. The greatest difference was observed between high and low deprivation, with an incidence rate ratio of 1.20 (95% CI: 1.14 to 1.27) among men and 1.21 (95% CI: 1.14 to 1.27) among women in 2014.

**Conclusions:**

There was a positive trend in the decline in age-adjusted type 2 diabetes incidence between 2014 and 2019. However, social inequality persisted with deprived groups at higher risk of type 2 diabetes. The level of inequality was comparable between men and women. Continued monitoring is essential to assess whether these short-term trends persist over time.

**STRENGTHS AND LIMITATIONS OF THIS STUDY**

- This study includes all persons in statutory health insurance, covering approximately 85% of the German population.
- This is the first study that reports temporal trends in sex-specific type 2 diabetes incidence by socioeconomic position in Germany.
- The observation period of the incidence rate is 6 years, which is too short to estimate long-term trends.
- The incidence of type 2 diabetes is probably underestimated as only information on diagnosed cases is available and people who did not make use of outpatient services in the respective calendar year are also not recorded in the database.

## Introduction

 The number of persons with type 2 diabetes continuously increased globally in recent decades, leading to a substantial disease and economic burden on individual and population level.^1^,^4^ In 2019, the global prevalence of diabetes was estimated at 463 million,^1^ which increased to 536.6 million persons in 2021.^5^

Besides the already high disease burden, a substantial increase is also expected in the future. Between 2015 and 2040, previous projections showed a substantial increase in the number of persons having type 2 diabetes in Germany,^6 7^ with a national prevalence of 9.48 in 2023.^8^ A major factor affecting current and future numbers of type 2 diabetes cases is the incidence of type 2 diabetes, that is, the number of new cases in relation to the size of the population without type 2 diabetes.^6 9^

It is well known that health is associated with indicators of socioeconomic position (SEP) and that socially deprived regions in general have higher prevalences and incidences of type 2 diabetes.^210^,^15^ Hering *et al*^8^ showed regional differences in the incidence and prevalence of type 2 diabetes at county level in Germany for the years 2011–2023: the highest values occurred in the eastern federal states and the lowest in the western federal states.^8^ These patterns are reflected in the pronounced socioeconomic deprivation, particularly in the north and northeast of Germany.^16^

The term ‘social deprivation’ refers to the degree of disadvantage for the population in a residential area, which results from a relative lack of socioeconomic and material resources and a comparatively high social spatial burden.^16 17^ For instance, Jacobs *et al*^11^ estimated that in 2012 the incidence of type 2 diabetes in Germany was more than twice as high among persons living in the most deprived regions compared with those living in the least deprived regions.^11^ The study by Reitzle *et al*^18^ also found substantial inequalities in the incidence of type 2 diabetes in Germany in 2021 depending on the German Index of Socioeconomic Deprivation (GISD).^18^ Although previous studies reported that the incidence of type 2 diabetes decreased in several high-income countries,^19^ including Germany,^9 18 20^ temporal trends in socioeconomic inequalities in type 2 diabetes incidence are currently unknown for Germany. Hence, we aimed to estimate trends in type 2 diabetes incidence by sex and SEP between 2014 and 2019.

## Research design and methods

### Research design and data source

To estimate trends in type 2 diabetes incidence stratified by sex and SEP between 2014 and 2019, we performed an ecological study based on ambulatory billing data aggregated on the level of all 401 German counties. The data were provided by the Central Research Institute for Ambulatory Health Care in Germany (Zi). To preserve the anonymity of the persons, the data containing the number of incident cases and the population at risk were aggregated by county, age and sex, with a minimum number of 30 persons in each age and sex stratum. The population at risk are persons without a type 2 diabetes diagnosis during the previous 3 years. On the national level, the data were available in twenty 5-year age groups (<5 years to ≥95 years) stratified by sex. Due to the fine-grained regional stratification into the 401 counties, age stratification was not possible on the county level. However, the number of persons at risk of incident type 2 diabetes could be provided in 5-year age groups, which enabled indirect age standardisation as described below. All data were available for each year between 2014 and 2019.

### Inclusion and exclusion criteria

The data include all persons in statutory health insurance (approximately 85% of the German population) who had at least one contact with a physician per year. Persons with invalid place of residence (county) or age >111 years were excluded. As in previous studies using these data, persons who have already been diagnosed with diabetes during the previous 3 years were excluded to exclude persons with prevalent type 2 diabetes.^9 21^

### Variables

Incident type 2 diabetes cases were identified based on the International Statistical Classification of Diseases and Related Health Problems, 10th revision (ICD-10), and as documented for billing purposes. Only confirmed diagnoses were considered. To reduce the probability of false-positive diagnoses, there had to be at least one further type 2 diabetes coding after the first coding of a type 2 diabetes.^9 21^ For a type 2 diabetes diagnosis, ICD-10 codes E11 (type 2 diabetes), E12 (malnutrition-related diabetes mellitus), E13 (other specified diabetes mellitus) and E14 (unspecified diabetes mellitus) were considered in certain combinations. Diagnoses E12-E14 were included because the number of type 2 diabetes diagnoses is probably underestimated if only E11 diagnoses are considered.^9 21^

Since the data do not contain information on SEP, we used the area-level GISD as a proxy variable for the individual SEP. The GISD is a publicly available index based on aggregated regional data and reflects the SEP on county level.^16 17^ The GISD combines information on education, employment and income with a number ranging between 0 (lowest deprivation, i.e., highest SEP) and 1 (highest deprivation, i.e., lowest SEP).

### Statistical analysis

To estimate the age-adjusted incidence of type 2 diabetes between 2014 and 2019, we used a mixed negative binomial regression model, including the GISD, sex and calendar year as independent variables, the observed number of type 2 diabetes cases as dependent variable and a random intercept for the counties to account for heterogeneity between and correlation within counties. For the random intercept, we chose an unstructured variance–covariance structure. The GISD was modelled linearly while calendar year was modelled as a natural cubic spline with two knots at equally spaced quantiles to allow for a non-linear association. The number of knots was chosen based on the best model fit according to the Bayesian information criterion. Sex was modelled as a categorical variable. We included a three-way interaction between GISD, sex and calendar year to allow for sex and calendar year-specific associations between the GISD and the type 2 diabetes incidence. To control for the effect of age, we included the expected number of type 2 diabetes cases in the counties as an offset term. The expected number of type 2 diabetes cases was calculated based on the national age-specific incidence in 2014 and the age-specific number of persons at risk in the counties. Including the logarithm of the expected number of cases as an offset term is equivalent to modelling the logarithm of the ratio of the observed and expected number of cases, which is also known as the age-standardised incidence ratio (SIR) or indirect age standardisation.^22^ The age-adjusted incidence was calculated by multiplying the SIR from the negative binomial regression model with the crude national incidence in 2014.^22^ We also report incidence rate ratios (IRRs) and corresponding 95% CIs from the regression model to compare the incidence between GISD groups. For this, we defined five equally sized groups based on the quintiles of the empirical GISD distribution and report the IRRs for the midpoint of these groups, respectively. All analyses were conducted with R (The R Foundation for Statistical Computing), V.4.3.1.

### Patient and public involvement

Patients and/or the public were not involved in the design, or conduct, or reporting, or dissemination plans of our research.

## Results

In total, approximately 63 million persons at risk were included in the analyses each year. An overview of the number of persons at risk and the incident type 2 diabetes cases by sex and quintiles of the GISD for the years 2014–2019 is given in table 1.

**Table 1:** Characteristics of the study population

	Quintile 1 (lowest deprivation)	Quintile 2	Quintile 3	Quintile 4	Quintile 5 (highest deprivation)	Total
Population at risk, n	75 615 269	76 024 675	75 721 029	74 853 215	78 679 196	380 893 384
Calendar year, n (%)						
2014	12 419 865 (19.9%)	12 491 121 (20.0%)	12 427 266 (19.9%)	12 194 582 (19.6%)	12 804 137 (20.5%)	62 336 971
2015	12 312 258 (19.6%)	12 646 372 (20.2%)	12 557 397 (20.0%)	10 649 568 (17.0%)	14 396 578 (23.0%)	62 562 173
2016	12 597 986 (19.9%)	12 456 661 (19.7%)	12 773 658 (20.2%)	12 615 142 (19.9%)	12 722 956 (20.1%)	63 166 403
2017	12 624 423 (19.8%)	12 794 122 (20.0%)	12 512 358 (19.6%)	13 042 179 (20.4%)	12 801 013 (20.0%)	63 774 095
2018	12 919 076 (19.9%)	12 817 017 (19.7%)	12 979 794 (20.0%)	13 054 809 (20.1%)	13 075 284 (20.2%)	64 845 980
2019	12 741 661 (19.8%)	12 819 382 (20.0%)	12 470 556 (19.4%)	13 296 935 (20.7%)	12 879 228 (20.0%)	64 207 762
Women, n (%)	41 791 281 (20.0%)	41 590 287 (19.9%)	41 180 387 (19.7%)	40 874 753 (19.6%)	42 842 036 (20.6%)	208 278 744
German Index of Socioeconomic Deprivation						
Mean (SD)	0.28 (0.11)	0.45 (0.04)	0.55 (0.03)	0.63 (0.03)	0.76 (0.07)	0.53 (0.17)
Incident type 2 diabetes cases, n	469 355 (17.1%)	514 249 (18.7%)	513 692 (18.7%)	578 748 (21.1%)	666 913 (24.3%)	2 742 957
Calendar year, n (%)						
2014	85 674 (17.9%)	90 591 (18.9%)	87 596 (18.3%)	100 953 (21.0%)	115 179 (24.0%)	479 993
2015	77 487 (16.7%)	87 163 (18.8%)	86 419 (18.7%)	85 925 (18.6%)	126 081 (27.3%)	463 075
2016	75 116 (16.4%)	82 067 (18.0%)	87 637 (19.2%)	100 790 (22.1%)	111 041 (24.3%)	456 651
2017	75 520 (17.0%)	83 098 (18.7%)	84 177 (19.0%)	97 630 (22.0%)	102 959 (23.2%)	443 384
2018	78 522 (17.6%)	84 414 (18.9%)	83 111 (18.6%)	95 555 (21.4%)	105 797 (23.7%)	447 399
2019	77 036 (17.0%)	86 916 (19.2%)	84 752 (18.7%)	97 895 (21.6%)	105 856 (23.4%)	452 455
Women, n (%)	232 873 (17.3%)	253 170 (18.7%)	250 804 (18.6%)	284 964 (21.1%)	327 052 (24.2%)	1 348 863

**Figure 1:**
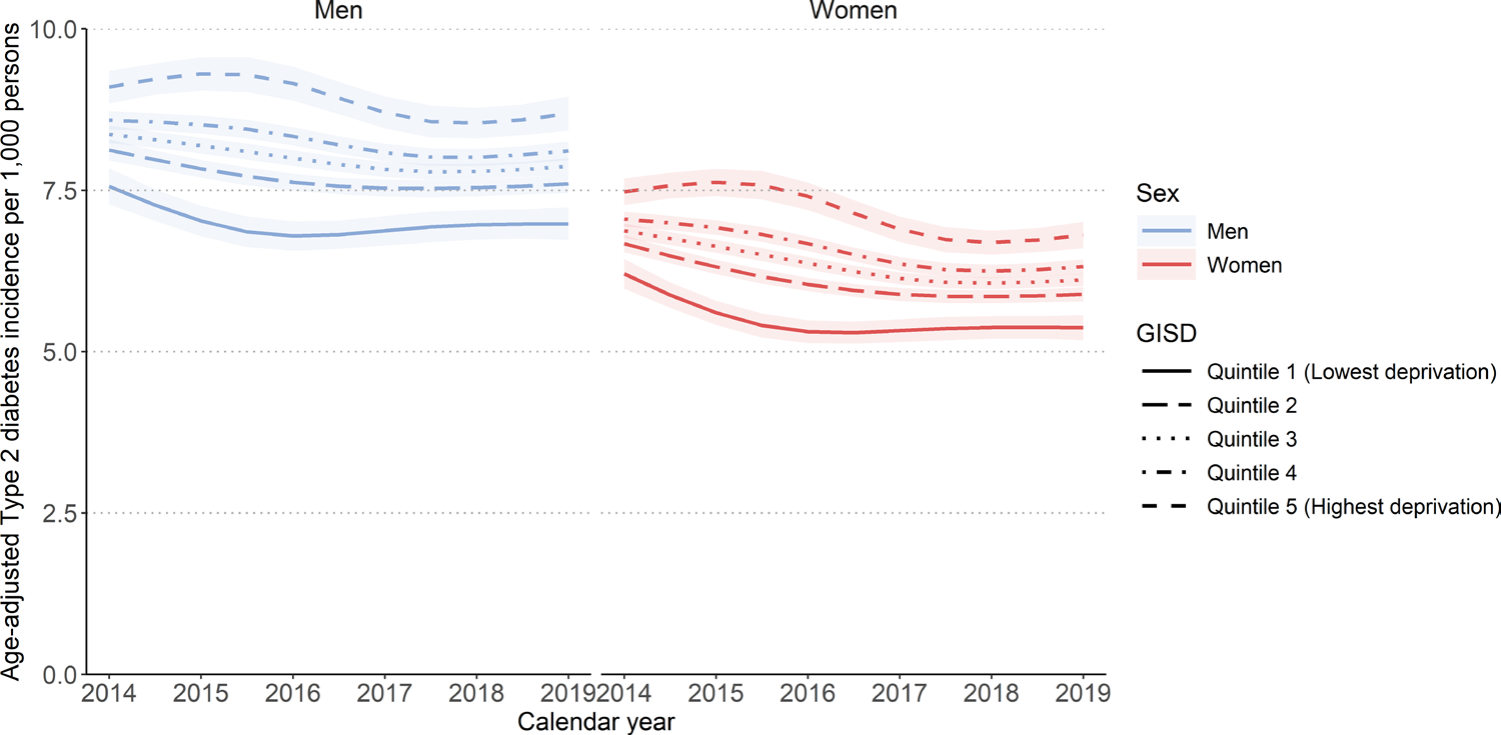
Age-adjusted incidence of type 2 diabetes (2014–2019) by quintiles of the German Index of Socioeconomic Deprivation (GISD). Results are based on a mixed negative binomial regression model. The model included GISD (linear), sex and calendar year (natural cubic spline with 3 degrees of freedom) as predictors, with the logarithm of expected cases as offset and counties as random intercepts. The outcome was the number of observed type 2 diabetes cases.

Figure 1 shows the estimated age-adjusted incidence per 1000 persons between 2014 and 2019 for each quintile of the empirical GISD distribution stratified by sex, based on the mixed negative binomial regression model. The lines refer to the model-based predicted values for the midpoints of five equally sized GISD groups (quintiles). The midpoints for the groups were 0.19, 0.44, 0.55, 0.64 and 0.84. Shaded areas indicate 95% CIs. The course of the age-adjusted incidence across the years was similar between both sexes and all GISD groups. The estimated age-adjusted incidence was lower in women than in men. The incidence decreased between 2014 and 2017 and plateaued thereafter. Increasing socioeconomic deprivation was associated with higher incidence, with the largest difference between the highest and lowest deprivation quintile. In the group with the highest incidence, that is, the group with the highest deprivation, the age-adjusted incidence decreased from 9.09 (95% CI: 8.85 to 9.35) per 1000 persons in 2014 to 8.65 (95% CI: 8.42 to 8.95) per 1000 persons in 2019 for men and from 7.47 (95% CI: 7.27 to 7.68) per 1000 persons in 2014 to 6.80 (95% CI: 6.59 to 7.01) per 1000 persons in 2019 for women.

**Figure 2:**
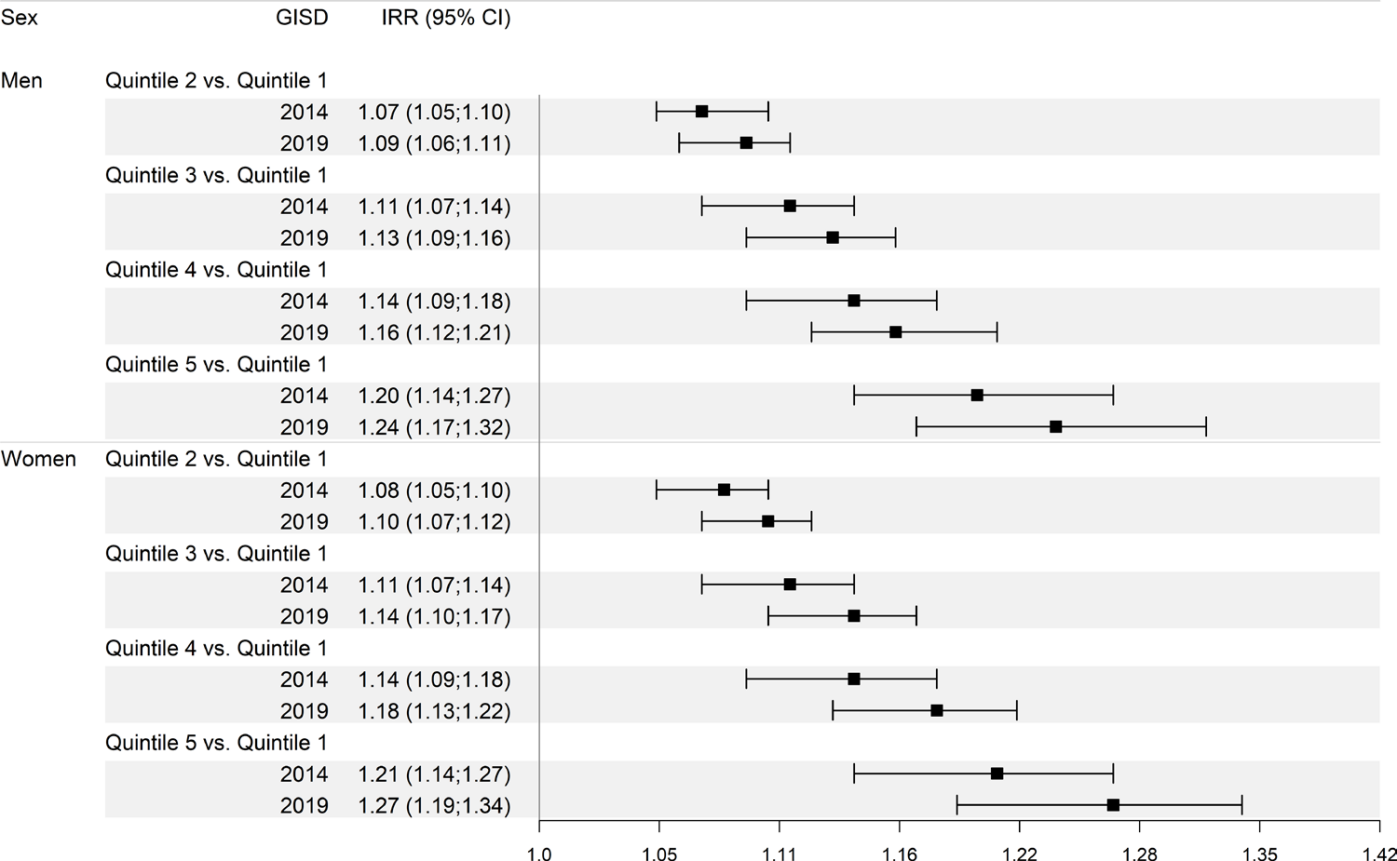
Incidence rate ratio comparing persons with lowest deprivation (quintile 1 of the German Index of Socioeconomic Deprivation (GISD)) to those with higher deprivation (quintiles 2–5), by sex and calendar years 2014 and 2019. Results are based on a mixed negative binomial regression model. The model included GISD (linear), sex and calendar year (natural cubic spline with 3 degrees of freedom) as predictors, with the logarithm of expected cases as offset and counties as random intercepts. The outcome was the number of observed type 2 diabetes cases.

In 2014, this difference amounted to an IRR of 1.20 (95% CI: 1.14 to 1.27) among men and 1.21 (95% CI: 1.14 to 1.27) among women (figure 2). The greater the deprivation level (quintiles 2–5 of the empirical GISD distribution) in relation to the lowest deprivation level (quintile 1 of the empirical GISD distribution), the greater the IRR. The IRR for all groups is slightly higher in 2019 than in 2014.

**Figure 3:**
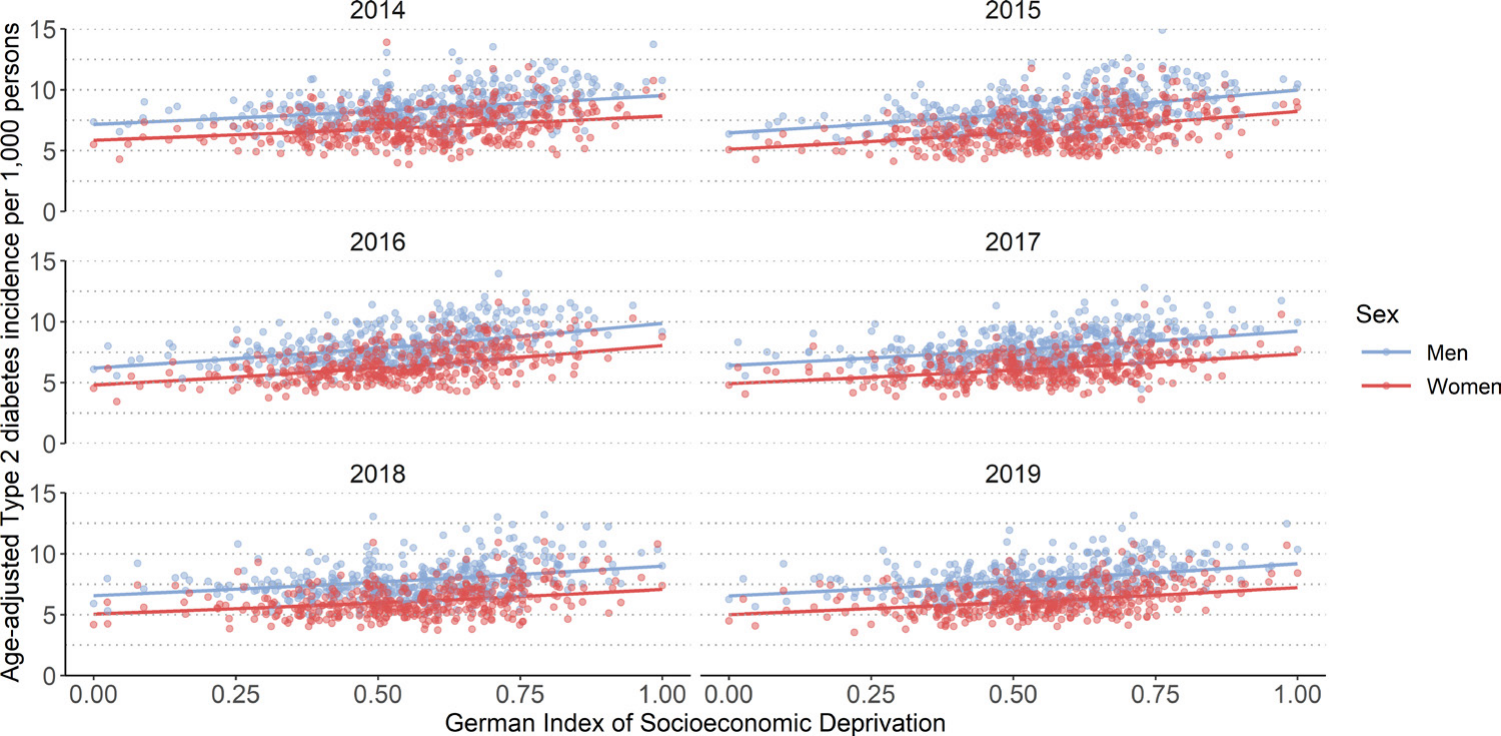
Age-adjusted incidence of type 2 diabetes by German Index of Socioeconomic Deprivation (GISD) from 2014 to 2019, stratified by sex. Results are based on a mixed negative binomial regression model. The model included GISD (linear), sex and calendar year (natural cubic spline with 3 degrees of freedom) as predictors, with the logarithm of expected cases as offset and counties as random intercepts. The outcome was the number of observed type 2 diabetes cases.

To provide a visual impression of the socioeconomic inequality in incidence along the whole range of the GISD, figure 3 shows the relationship between the continuous GISD and the age-adjusted incidence. Similar to figure 1, the age-adjusted incidence per 1000 persons was consistently lower in women than in men. Moreover, the age-adjusted incidence of the counties shows a rather large range between approximately 4 per 1000 and 13 per 1000.

**Figure 4:**
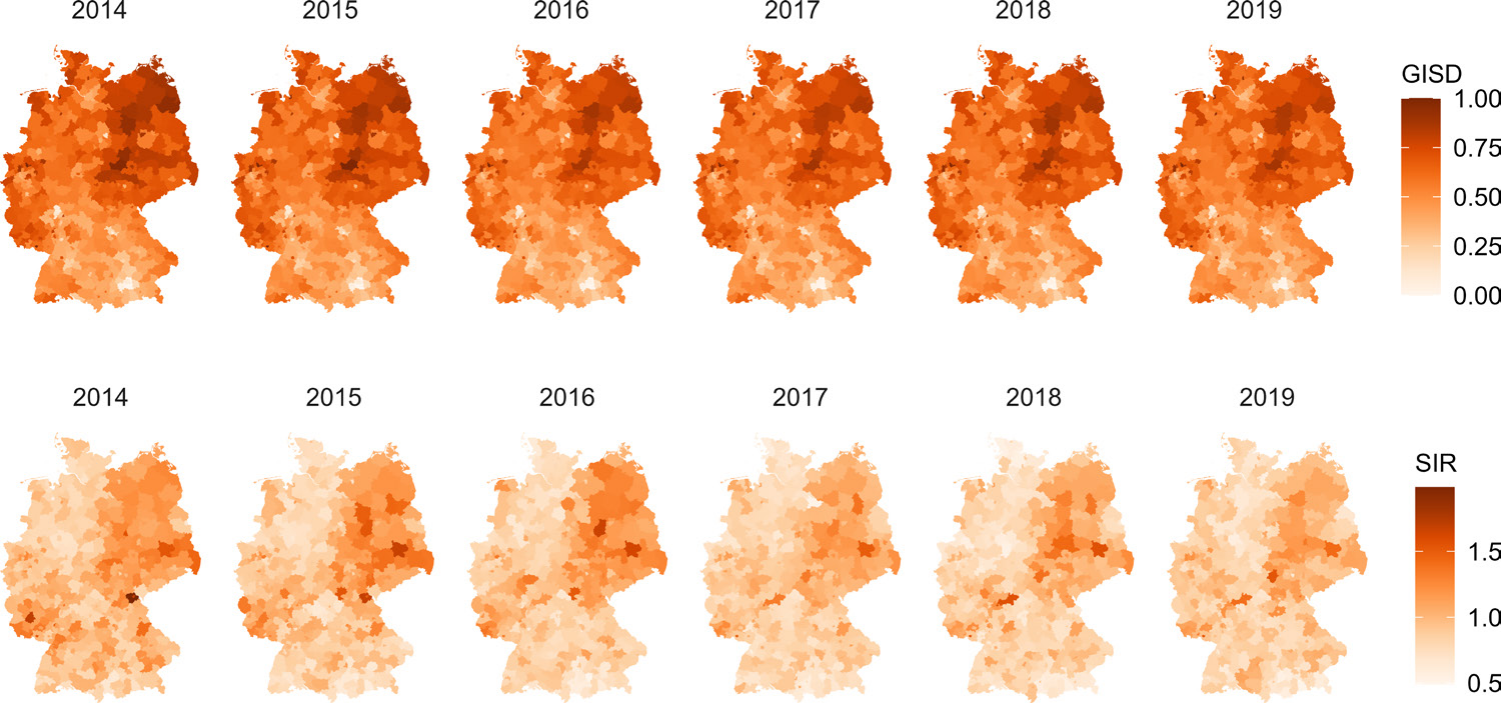
Distribution of the German Index of Socioeconomic Deprivation (GISD) and the age-standardised Incidence Ratio (SIR) in Germany between 2014 and 2019.

To complement this, figure 4 maps the spatial distribution of the SIR and the GISD across counties. The map reveals clear regional patterns: both SIR and socioeconomic deprivation tend to be higher in eastern Germany. While the east–west gradient in deprivation is well-established, the spatial visualisation highlights its potential relevance for the geographic distribution of type 2 diabetes incidence. In addition, the results in figure 3 show that the linearity assumption made between the age-adjusted incidence and the GISD in the mixed negative binomial regression model seems reasonable. The age-adjusted incidence increased with increasing GISD, that is, with lower SEP.

## Discussion

### Summary findings

Based on data from approximately 63 million persons insured by statutory health insurance, we estimated trends in the incidence of type 2 diabetes stratified by sex and SEP between 2014 and 2019.

The analyses showed clear differences in the incidence depending on the GISD in both men (IRR in 2014 of 1.20, 95% CI: 1.14 to 1.27) and women (IRR in 2014 of 1.21, 95% CI: 1.14 to 1.27), comparing the highest deprivation group (GISD quintile 5) with the lowest deprivation group (GISD quintile 1). Despite decreasing type 2 diabetes incidence in all GISD groups, the differences between GISD groups persisted over the entire study period and showed no clear upward or downward trend.

In the group with the highest incidence, that is, the group with the highest deprivation, the age-adjusted incidence decreased for both sexes. We also observed that the age-adjusted incidence among men was higher than among women, whereas the sex-specific differences by SEP were similar.

### Consistency with plausible theoretical justifications of the study findings

Our findings are in line with previous studies reporting decreases in type 2 diabetes incidence in recent years and socioeconomic inequalities in type 2 diabetes incidence.^9 11 12 18 20^ For instance, Reitzle *et al*^18^ estimated the incidence of type 2 diabetes before and after the COVID-19 pandemic in Germany. They found a decrease in the age-standardised incidence from 7.4 per 1000 persons in 2015 to 6.7 per 1000 persons in 2017, which plateaued until 2019 and increased again in 2021 to the level of 2015. The type 2 diabetes incidence among women was lower than among men. Reitzle *et al*^18^ also analysed the type 2 diabetes incidence by GISD groups for the years 2019–2021 and found inequalities that were similar to our results. However, trends in inequalities over several years were not reported.

A recent study from France revealed that the age-adjusted incidence in the least deprived regions was lower (8.6 per 1000 persons for men, 5.0 per 1000 persons for women) compared with the most deprived regions (12.7 per 1000 persons for men, 8.3 per 1000 persons for women) in 2020.^12^ Deprivation was measured with the French deprivation index, which is an area-based deprivation indicator. The analysis was also based on billing data and found that the age-adjusted incidence in France decreased between 2012 and 2020. It was also reported that the type 2 diabetes incidence was higher in men compared with women. The greatest difference to our analyses is that the French study only included persons above the age of 45 years, whereas we considered a much broader age range.

### Implications for future research and interventions for Germany

Reasons for the decreasing type 2 diabetes incidence in Germany cannot be precisely determined. One reason could be the higher consumption of vegetables and a reduced consumption of red meat, especially pork, and alcohol between 1991 and 2018.^23^ On the other hand, national data on outpatient services show that the prevalence of diagnosed obesity increased significantly between 2009 and 2018.^24^ Despite decreasing trends in type 2 diabetes incidence, developments should continue to be monitored, particularly with regard to the COVID-19 pandemic. Although type 2 diabetes incidence continued to decrease in the first year of the pandemic (2020), it increased again in 2021.^18^ Besides overall trends in incidence, socioeconomic inequalities should be closely monitored as well since our results show no improvement in terms of inequality reduction between 2014 and 2019. In addition to a trend analysis, a cluster analysis as in Ganasegeran *et al*^25^ could be performed to identify possible clusters for targeted interventions. Socioeconomic inequalities in health are not only relevant with regard to the incidence of type 2 diabetes. They remain a major problem for overall public health in Germany and have been consistently documented for various health outcomes. A recent nationwide study by Hoebel *et al*^26^ showed that life expectancy is substantially lower in socioeconomically deprived areas compared with more deprived regions. These regional disparities underscore the importance of structural and contextual determinants of health.^26^ Therefore, measures should be intensified to minimise these inequalities. These measures could include interventions targeted at persons in low SEP as well as more context-oriented approaches. For example, taxes on different foods could be adjusted^27 28^ to encourage healthier lifestyles, more green spaces could be created in deprived areas^29^ and projects aiming an improved access to medical care and health education in deprived regions such as the ‘Gesundheitskioske in HH Billstedt’^30^ could also be introduced. Besides prevention, our results indicate that the demand for diabetes care is higher in more deprived regions. One problem might be that the density of medical practices is lower in deprived regions compared with less deprived regions in Germany.^31 32^ If the observed inequalities continue to persist or even increase in future, medical care for persons with diabetes in deprived regions could become increasingly problematic, which, once again, highlights the importance of timely monitoring and counteracting the socioeconomic inequalities in type 2 diabetes incidence.

### Strengths and limitations

Our study has several strengths. First, it was based on all persons in statutory health insurance, covering approximately 85% of the German population, while previous studies relied on smaller samples of statutory health insurance data.^12 18 20^ Second, this is the first study that reports temporal trends in sex-specific type 2 diabetes incidence by SEP. The advantage offered by the GISD is that it is possible to analyse health inequalities in Germany even if the data do not contain information on SEP at the individual level. However, using the GISD implies that the county-specific GISD is a good proxy for the individual SEP, which might not always be the case.

The following further limitations should be taken into account when interpreting the results. Although the observation period covers 6 years, it is still too short to estimate long-term trends. In studies from the USA, the UK and the Netherlands, for example, data sources are available that allow the incidence of type 2 diabetes to be monitored over several decades.^33^,^35^ In addition, the exclusion of persons who did not use any outpatient services during a calendar year might have led to selection bias. For instance, the incidence rate would be underestimated if diabetes risk was associated with the probability to visit a physician at least once a year. Furthermore, the type 2 diabetes incidence is probably underestimated because information was only available for diagnosed type 2 diabetes. The diagnosis of type 2 diabetes is affected by several external factors. These include reimbursement by the health insurance companies and public awareness of the disease. Moreover, the database depends on the coding behaviour of the physician. It can therefore not be ruled out that temporal changes in the incidence of type 2 diabetes are based on adjustments in the documentation, for example, for administrative reasons. In addition, our data are not representative for patients with private health insurance as our data only include those with statutory health insurance. A final limitation is that our study design only examines distal determinants, the SEP, which indirectly affect the incidence of type 2 diabetes. Proximal determinants, such as individual diet or physical activity, cannot be investigated directly with our study design as we do not have individual data available. Nonetheless, the consideration of distal determinants remains essential as they shape the structural conditions under which proximal risk factors develop. While proximal factors directly influence disease onset, distal factors—such as education and income or neighbourhood—systematically affect health behaviours and access to resources. Focusing solely on proximal determinants risks overlooking these upstream causes of health inequalities.^36^,^38^

## Conclusions

In summary, our findings suggest that the incidence of type 2 diabetes in Germany decreased from 2014 to 2019. Differences were observed both by sex and SEP. Socioeconomic inequalities in the incidence persisted between 2014 and 2019. Persons with a low SEP consistently exhibited a higher incidence of type 2 diabetes compared with those with a high SEP. It is important to continue monitoring the development to determine whether these short-term trends will continue in the future.^25^ Further investigations of causes and potential measures to address socioeconomic disparities in the incidence of type 2 diabetes should be conducted.

## Data Availability

Data are available upon reasonable request. Data may be obtained from a third party and are not publicly available.
