# Supplementary material for: Incidence of type 2 diabetes by socioeconomic deprivation in Germany between 2014 and 2019: an ecological study
Source: BMJ Open. 2025 Jul 16;15(7):e094824. doi: 10.1136/bmjopen-2024-094824 (PMC12273173; doi:10.1136/bmjopen-2024-094824)
Supplement: Supplementary file 1 [file bmjopen-2024-094824.reviewer_comments.pdf]

PEER REVIEW HISTORY

BMJ Open publishes all reviews undertaken for accepted manuscripts. Reviewers are asked to complete a checklist review form and are provided with free text boxes to elaborate on their assessment. These free text comments are reproduced below.

ARTICLE DETAILS

Title (Provisional)

Incidence of Type 2 diabetes by socioeconomic deprivation in Germany between 2014 and 2019: An ecological study

Authors

Piedboeuf, Katharina; Hering, Ramona; Schulz, Mandy; Mackowiak, Malwina; Brinks, Ralph; Kuss, Oliver; Hoyer, Annika; Tönnies, Thaddäus

VERSION 1 - REVIEW

**Reviewer**	**1**
**Name**	**Moinuddin, Mohammed**
**Affiliation**	**University of Lancashire, School of Medicine and Dentistry**
**Date**	**04-Nov-2024**
**COI**	**None**

Results from an ecological study were presented estimating the incidence of type 2 diabetes. An interesting research topic, statistical analysis was sound however some minor modifications would increase the readability and make it informative. My specific observations are below.

Comment 1 – The ICD-10 codes e12-E14 were not specified as type 2. Why is this justified to include these codes as a diagnosing code for type 2 diabetes?

Comment 2 – Give an idea of how big a GISD area is. If the area is too big and it is possible that there could be people with different SEP levels in the same area, then it would be difficult to justify the use of area-level SEP measures.

Table 1 – The incidence rates were presented as column percentages which is not a useful measure as it will depend on population size. Rather a row percentage would provide some level of comparison of T2D incidence by deprivation quintile.

Figure legends are too large. Instead of describing full results in the legends, it would be good to describe them in the result section.

**Reviewer**	**2**
**Name**	**Ganasegeran, Kurubaran**
**Affiliation**	**Ministry of Health Malaysia, Public Health Unit**
**Date**	**02-Dec-2024**
**COI**	**None**

I have evaluated the paper by Piedboeuf et al for their work on "Incidence of Type 2 diabetes by socioeconomic deprivation in Germany between 2014 and 2019: An ecological study." While the work has some interesting novel interpretations, the paper requires some revisions before being accepted for publication.

1. The abstract lacks the style as required by the journal. Please structure into the following subsections: Objective, design, primary outcome, study population and setting, results, conclusion.

2. The introduction is relatively short and requires some extensions. I suggest that authors describe the problem statement in detail, supported with evidence from a more detailed literature review, pointing to the the needs-gaps, making comparisons to other countries or settings, and then justify the needs for conducting this study.

3. Authors should note that this is an ecological study, hence it is worthwhile to describe the importance of the design in terms of determining the distal determinants as compared to the proximal determinants of socioeconomic deprivation from the literature. This could be supported by a simple conceptual diagram.

4. In it's present form, authors only conducted a trend analysis. While this was worthwhile to see the distribution and patterns of type 2 diabetes incidence, it would be more meaningful if authors could map those rates by county shapefile to see possible clusters for targeted interventions. See the work https://doi.org/10.1007/s44197-023-00185-2 , also it would be worthwhile to see the quintile maps and it is feasible to do considering authors have all the relevant sources to map one based on their study objectives.

5. Following the above comment, authors could provide further extensions on the description of the results and discussions on how place (certain counties or regions) in Germany may have affected the patterns and trends of type 2 diabetes incidence.

6. The discussion should be structured as core summary findings, consistency with plausible theoretical justifications of the study findings, implications for future research and interventions for Germany.

7. Most of the references are relatively old. Please update or add newer ones.

**Reviewer**	**3**

**Name**	**ghammari, fawzieh**
**Affiliation**	**Tabriz University of Medical Sciences**
**Date**	**18-Apr-2025**
**COI**	**None**

Abstract

Conclusions

Please don't present results in conclusions, but your conclusions of the study.

Introduction

Please present more information about the prevalence of T2D in Germany. Additionally, be clearer about inequalities in Germany counties.

Method

Please mention inclusion and exclusion criteria in a separate section.

Please provide strengths and limitations as well as conclusions in separate sections

VERSION 1 - AUTHOR RESPONSE

Reviewer: 1

Results from an ecological study were presented estimating the incidence of type 2 diabetes. An interesting research topic, statistical analysis was sound however some minor modifications would increase the readability and make it informative. My specific observations are below.

1. Comment 1 – The ICD-10 codes e12-E14 were not specified as type 2. Why is this justified to include these codes as a diagnosing code for type 2 diabetes?
   Reply: *As in previous analyses, we used the diagnostic criteria by Goffier et al. (DOI: 10.20364/VA-17.03), where it was shown that Type 2 diabetes is strongly underestimated if unspecific diabetes diagnoses are not considered. This is the reason why we and many other studies assigned the codes E12-E14 to Type 2 diabetes.*

2. Comment 2 – Give an idea of how big a GISD area is. If the area is too big and it is possible that there could be people with different SEP levels in the same area, then it would be difficult to justify the use of area-level SEP measures.
   Reply: *Thank you very much for raising this important issue. As is the case with all area-based measures of SEP, they are an average and therefore an imperfect representation of the individual SEP. In our case, the GISD is based on the 401 counties in Germany which vary in population size between 35,000 and 3.8 million. Although this is a considerable variation, we think the GISD at this regional level serves as a reasonable proxy for individual SEP. This is supported by the fact that this index is also used by the official health surveillance in Germany (DOI: 10.1007/s00103-024-03862-0). Nevertheless, we acknowledge that this is a limitation of our study, as is also mentioned in our manuscript:*

   > *"The advantage offered by the GISD is that it is possible to analyze health inequalities in Germany even if the data do not contain information on SEP at the individual level. However, using the GISD implies*

that the county-specific GISD is a good proxy for the individual SEP, which might not always be the case."

3. Table 1 – The incidence rates were presented as column percentages which is not a useful measure as it will depend on population size. Rather a row percentage would provide some level of comparison of T2D incidence by deprivation quintile.
   Reply: *Thank you for the helpful comment. We have revised Table 1 to present row percentages instead of column percentages, allowing for a clearer comparison of T2D incidence across deprivation quintiles:*

Table 1. Characteristics of study population.

	Quintile 1 (Lowest deprivation)	Quintile 2	Quintile 3	Quintile 4	Quintile 5 (Highest deprivation)	Total
**Population at risk**, n	75,615,269	76,024,675	75,721,029	74,853,215	78,679,196	380,893,384
**Calendar year**, n (%)						
2014	12,419,865 (19.9%)	12,491,121 (20.0%)	12,427,266 (19.9%)	12,194,582 (19.6%)	12,804,137 (20.5%)	62,336,971
2015	12,312,258 (19.6%)	12,646,372 (20.2%)	12,557,397 (20.0%)	10,649,568 (17.0%)	14,396,578 (23.0%)	62,562,173
2016	12,597,986 (19.9%)	12,456,661 (19.7%)	12,773,658 (20.2%)	12,615,142 (19.9%)	12,722,956 (20.1%)	63,166,403
2017	12,624,423 (19.8%)	12,794,122 (20.0%)	12,512,358 (19.6%)	13,042,179 (20.4%)	12,801,013 (20.0%)	63,774,095
2018	12,919,076 (19.9%)	12,817,017 (19.7%)	12,979,794 (20.0%)	13,054,809 (20.1%)	13,075,284 (20.2%)	64,845,980
2019	12,741,661 (19.8%)	12,819,382 (20.0%)	12,470,556 (19.4%)	13,296,935 (20.7%)	12,879,228 (20.0%)	64,207,762
**Women**, n (%)	41,791,281 (20.0%)	41,590,287 (19.9%)	41,180,387 (19.7%)	40,874,753 (19.6%)	42,842,036 (20.6%)	208,278,744
**GISD**						
Mean (SD*)	0.28 (0.11)	0.45 (0.04)	0.55 (0.03)	0.63 (0.03)	0.76 (0.07)	0.53 (0.17)
**Incident Type 2 diabetes cases**, n	469,355 (17.1 %)	514,249 (18.7 %)	513,692 (18.7 %)	578,748 (21.1 %)	666,913 (24.3 %)	2,742,957
**Calendar year**, n (%)						
2014	85,674 (17.9 %)	90,591 (18.9 %)	87,596 (18.3 %)	100,953 (21.0 %)	115,179 (24.0 %)	479,993
2015	77,487 (16.7 %)	87,163 (18.8 %)	86,419 (18.7 %)	85,925 (18.6 %)	126,081 (27.3 %)	463,075
2016	75,116 (16.4 %)	82,067 (18.0 %)	87,637 (19.2 %)	100,790 (22.1 %)	111,041 (24.3 %)	456,651
2017	75,520 (17.0 %)	83,098 (18.7 %)	84,177 (19.0 %)	97,630 (22.0 %)	102,959 (23.2 %)	443,384
2018	78,522 (17.6 %)	84,414 (18.9 %)	83,111 (18.6%)	95,555 (21.4 %)	105,797 (23.7 %)	447,399
2019	77,036 (17.0 %)	86,916 (19.2 %)	84,752 (18.7 %)	97,895 (21.6 %)	105,856 (23.4 %)	452,455
**Women**, n (%)	232,873 (17.3 %)	253,170 (18.7 %)	250,804 (18.6 %)	284,964 (21.1 %)	327,052 (24.2 %)	1,348,863

4. Figure legends are too large. Instead of describing full results in the legends, it would be good to describe them in the result section.
   Reply: *Thank you for addressing this point. We added shorter versions for the figure legends and applied the recommended changes in the results section:*

   ***"Figure 1:** Age-adjusted incidence of Type 2 diabetes (2014–2019) by quintiles of the German Index of Socioeconomic Deprivation (GISD).*
   Results are based on a mixed negative binomial regression model. The model included GISD (linear), sex, and calendar year (natural cubic spline with 3 degrees of freedom) as predictors, with the logarithm of expected cases as offset and counties as random intercepts. The outcome was the number of observed Type 2 diabetes cases."

*"**Figure 2:** Incidence rate ratio (IRR) comparing persons with lowest deprivation (Quintile 1 of the German Index of Socioeconomic Deprivation, GISD) to those with higher deprivation (Quintiles 2–5), by sex and calendar years 2014 and 2019.*
Results are based on a mixed negative binomial regression model. The model included GISD (linear), sex, and calendar year (natural cubic spline with 3 degrees of freedom) as predictors, with the logarithm of expected cases as offset and counties as random intercepts. The outcome was the number of observed Type 2 diabetes cases."

*"**Figure 3:** Age-adjusted incidence of Type 2 diabetes by GISD from 2014 to 2019, stratified by sex. Results are based on a mixed negative binomial regression model. The model included GISD (linear), sex, and calendar year (natural cubic spline with 3 degrees of freedom) as predictors, with the logarithm of expected cases as offset and counties as random intercepts. The outcome was the number of observed Type 2 diabetes cases."*

Description of figures in Results section (example):
"The lines refer to the model-based predicted values for the midpoints of five equally sized GISD groups (quintiles). The midpoints for the groups were 0.19, 0.44, 0.55, 0.64 and 0.84. Shaded areas indicate 95%-confidence intervals."

Reviewer: 2

I have evaluated the paper by Piedboeuf et al for their work on "Incidence of Type 2 diabetes by socioeconomic deprivation in Germany between 2014 and 2019: An ecological study." While the work has some interesting novel interpretations, the paper requires some revisions before being accepted for publication.

1. The abstract lacks the style as required by the journal. Please structure into the following subsections: Objective, design, primary outcome, study population and setting, results, conclusion.
   Reply: *We have revised the abstract to follow the structured format required by the journal, including the subsections: Objective, Design, Setting, Participants, Primary and secondary outcome measures, Results, and Conclusion:*

   *"**Objective***
   To estimate Type 2 diabetes incidence trends by sex and socioeconomic position (SEP) and evaluate trends in SEP-related inequalities in incidence.

   Design
   Ecological study using ambulatory claims data and regression-based modelling.

   Setting
   All 401 counties in Germany, covering the entire country.

   Participants
   All individuals with statutory health insurance (~85% of the population). Incident cases of Type 2 diabetes were identified annually from 2014 to 2019 using ICD-10 codes.

   Primary and secondary outcome measures
   Incident Type 2 diabetes at the county level, adjusted for age and modelled using a mixed negative binomial regression. SEP was measured using the German Index of Socioeconomic Deprivation (GISD), and a random intercept accounted for county-level heterogeneity.

Results
The incidence of Type 2 diabetes decreased between 2014 and 2017 and plateaued thereafter. Trends were similar between sexes and deprivation levels. The greatest difference was observed between high and low deprivation with an incidence rate ratio of 1.20 (95% confidence interval (CI): 1.14-1.27) among men and 1.21 (95% CI: 1.14-1.27) among women in 2014.

Conclusions
There was a positive trend in the decline in age-adjusted Type 2 diabetes incidence between 2014 and 2019. However, social inequality persisted with deprived groups at higher risk of Type 2 diabetes. The level of inequality was comparable between men and women. Continued monitoring is essential to assess whether these short-term trends persist over time."

2. The introduction is relatively short and requires some extensions. I suggest that authors describe the problem statement in detail, supported with evidence from a more detailed literature review, pointing to the needs-gaps, making comparisons to other countries or settings, and then justify the needs for conducting this study.
<u>Reply:</u> *Thank you very much for the suggestion. We extended the introduction accordingly. The corresponding part now reads:*

> *"Besides the already high disease burden, a substantial increase is also expected in the future. Between 2015 and 2040, previous projections showed a substantial increase in the number of persons having Type 2 diabetes in Germany (DOI: 10.1111/dme.13902, DOI: 10.3389/fepid.2025.1388189), with a national prevalence of 9,48 in 2023 (DOI: 10.20364/VA-24.02)."*

> *"Hering et al. (2024) showed regional differences in the incidence and prevalence of Type 2 diabetes at county level in Germany for the years 2011 to 2023: the highest values occurred in the eastern federal states and the lowest in the western federal states (DOI: 10.20364/VA-24.02). These patterns are reflected in the pronounced socioeconomic deprivation, particularly in the north and north-east of Germany (DOI: 10.25646/10640)."*

3. Authors should note that this is an ecological study, hence it is worthwhile to describe the importance of the design in terms of determining the distal determinants as compared to the proximal determinants of socioeconomic deprivation from the literature. This could be supported by a simple conceptual diagram.
<u>Reply:</u> *Thank you for addressing this point. We have added a paragraph in the Discussion that explicitly explains the relevance of using an ecological design to examine distal determinants (e.g., neighborhood-level characteristics, policy context) rather than proximal determinants (e.g., individual-level behaviors or exposures). The corresponding part reads:*

> *"A final limitation is that our study design only examines distal determinants, the SEP, which indirectly affect the incidence of Type 2 diabetes. Proximal determinants, such as individual diet or physical activity, cannot be investigated directly with our study design, as we do not have individual data available. Nonetheless, the consideration of distal determinants remains essential, as they shape the structural conditions under which proximal risk factors develop. While proximal factors directly influence disease onset, distal factors—such as education and income or neighborhood—systematically affect health behaviours and access to resources. Focusing solely on proximal determinants risks overlooking these upstream causes of health inequalities (DOI: 10.1186/s12963-020-00244-x, DOI: 10.2105/AJPH.91.11.1783, DOI: 10.1002/ijop.12928)."*

From our perspective, summarizing this complex issue in a meaningful and scientifically valid diagram would be the results of a study on its own. We therefore would like to refrain from adding a diagram to the discussion of our manuscript.

4. In its present form, authors only conducted a trend analysis. While this was worthwhile to see the distribution and patterns of Type 2 diabetes incidence, it would be more meaningful if authors could map those rates by county shapefile to see possible clusters for targeted interventions. See the work DOI: 10.1007/s44197-023-00185-2, also it would be worthwhile to see the quintile maps and it is feasible to do considering authors have all the relevant sources to map one based on their study objectives.
   Reply: *Thank you very much for this helpful suggestion. We now include maps of the GISD and the standardized incidence ratio in the manuscript (Figure 4). For future analyses and as a discussion point for the manuscript, we also address the opportunities of cluster analysis:*

   Update in the discussion:

   > *"In addition to a trend analysis, a cluster analysis as in Ganasegeran et al. could be performed to identify possible clusters for targeted interventions (DOI:* 10.1007/s44197-023-00185-2*)."*

5. Following the above comment, authors could provide further extensions on the description of the results and discussions on how place (certain counties or regions) in Germany may have affected the patterns and trends of type 2 diabetes incidence.
   Reply: *We thank the reviewer for this valuable comment. In response, we expanded the discussion section to include a more detailed reflection on regional and structural health inequalities in Germany. We emphasize that socioeconomic inequalities are not only relevant to Type 2 diabetes incidence but represent a broader and persistent public health issue.*

   Update in result section:

   > *"To complement this, Figure 4 maps the spatial distribution of the standardized incidence ratio (SIR) and the GISD across counties. The map reveals clear regional patterns: both SIR and socioeconomic deprivation tend to be higher in eastern Germany. While the east–west gradient in deprivation is well-established, the spatial visualization highlights its potential relevance for the geographic distribution of Type 2 diabetes incidence."*

   Update in the discussion:

   > *"Socioeconomic inequalities in health are not only relevant with regard to the incidence of Type 2 diabetes. They remain a major problem for overall public health in Germany and have been consistently documented for various health outcomes. A recent nationwide study by Hoebel et al. showed that life expectancy is substantially lower in socioeconomically deprived areas compared to more deprived regions. These regional disparities underscore the importance of structural and contextual determinants of health (DOI:* 10.25646/13026*)."*

6. The discussion should be structured as core summary findings, consistency with plausible theoretical justifications of the study findings, implications for future research and interventions for Germany.
   Reply: *Thank you for pointing this out, we have restructured the discussion accordingly.*

7. Most of the references are relatively old. Please update or add newer ones.
   Reply: *Thank you very much for this suggestion. We added references 4 (Bommer et al., 2018, DOI: 10.1016/S2213-8587(17)30097-9), 15 (Darvishi et al., 2024, DOI: 10.1186/s12889-024-18452-7) and 35 (Overbeek et al., 2024 DOI: 10.2147/DMSO.S445288) to the discussion of the manuscript.*

Reviewer: 3

1. Abstract, Conclusions, Please don't present results in conclusions, but your conclusions of the study.

Reply: *Thank you for your helpful comment. In accordance with your suggestion, we have revised the* Conclusions *section to remove detailed results and instead present the overarching conclusions and implications of our findings.*

> *"There was a positive trend in the decline in age-adjusted Type 2 diabetes incidence between 2014 and 2019. However, social inequality persisted and deprived groups remained at higher risk of Type 2 diabetes. The level of inequality was comparable between men and women. Continued monitoring is essential to assess whether these short-term trends persist over time."*

2. Introduction, Please present more information about the prevalence of T2D in Germany. Additionally, be clearer about inequalities in Germany counties.
   Reply: *We have revised the* Introduction *to include more detailed information on the prevalence of Type 2 diabetes in Germany, based on the most recent national reports. Additionally, we clarified the description of social and regional inequalities, with a specific focus on disparities between German counties, to better contextualize the study's objectives.*

   Update in the introduction:
   > *"Besides the already high disease burden, a substantial increase is also expected in the future. Between 2015 and 2040, previous projections showed a substantial increase in the number of persons having Type 2 diabetes in Germany (DOI: 10.1111/dme.13902, DOI: 10.3389/fepid.2025.1388189), with a national prevalence of 9,48 % in 2023 (DOI: 10.20364/VA-24.02)."*

   Update about inequalities in Germany counties:
   > *"Hering et al. (2024) showed regional differences in the incidence and prevalence of Type 2 diabetes at county level in Germany for the years 2011 to 2023: the highest values occurred in the eastern federal states and the lowest in the western federal states (DOI: 10.20364/VA-24.02). These patterns are reflected in the pronounced socioeconomic deprivation, particularly in the north and north-east of Germany (DOI: 10.25646/10640). "*

3. Method, Please mention inclusion and exclusion criteria in a separate section.
   Reply: *We added a separate section for this issue.*

4. Please provide strengths and limitations as well as conclusions in separate sections
   Reply: *We added a separate section for this issue.*

VERSION 2 - REVIEW

**Reviewer**	**2**
**Name**	**Ganasegeran, Kurubaran**
**Affiliation**	**Ministry of Health Malaysia, Public Health Unit**
**Date**	**26-Jun-2025**
**COI**	

Thank you for your revisions. I have no further comments
